# Supplementary material for: Nanoscale membranes that chemically isolate and electronically wire up the abiotic/biotic interface
Source: Nat Commun. 2018 Jun 11;9:2263. doi: 10.1038/s41467-018-04707-6 (PMC5995903; doi:10.1038/s41467-018-04707-6)
Supplement: Supplementary file 1 — Supplementary Information [file 41467_2018_4707_MOESM1_ESM.pdf]

SUPPLEMENTARY INFORMATION

Nanoscale membranes that chemically isolate and electronically wire up the abiotic/biotic interface

Cornejo et al.

Supplementary Methods

Synthesis of wire molecules

For the purpose of the Supplementary Information document, more descriptive designations are used for PV3 wire molecules with different substituents, shown in the CHEMDRAW structures below:

4,4'-((1*Z*,1'*Z*)-1,4-phenylenebis(2-cyanoethene-2,1-diyl))bis(3-nitrobenzoic acid):

PV3_CN_NO_2_CO_2H (abbreviated PV3 in the main text)

4,4'-((1*Z*,1'*Z*)-1,4-phenylenebis(2-cyanoethene-2,1-diyl))bis(*N*-(1,3-dihydroxy-2-(hydroxymethyl)propan-2-yl)-3-nitrobenzamide): **PV3_CN_NO_2_tripod**

4,4'-((1*Z*,1'*Z*)-1,4-phenylenebis(2-cyanoethene-2,1-diyl))bis(3-nitro-*N*-phenylbenzamide):

PV3_CN_NO_2_aniline

Cesium 4-((*E*)-4-((*E*)-4-((1,3-dihydroxy-2-(hydroxymethyl)-propan-2-yl)carbamoyl)-styryl) styryl) benzenesulfonate: **PV3_SO_3^-_tripod**

PV3_CN_NO_2_CO_2H wire synthesis. The synthesis strategy is based on Knoevenagel condensation of *p*-bis(cyanomethyl) benzene with 4-formyl-3-nitrobenzoic acid[1].

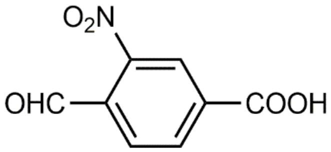

4-formyl-3-nitrobenzoic acid. 1.26 g (6 mmol) methyl 4-formyl-3-nitrobenzoate was dissolved in 60 mL methanol/water (1:1) solution of 1 M sodium hydroxide, deaerated with nitrogen and stirred at room temperature for 5 hours. After washing with ethyl acetate to remove the unreacted ester, the aqueous phase was adjusted to pH ~1.5 by 1N hydrochloric acid and then extracted with ethyl acetate. The ethyl acetate phase was dried over magnesium sulfate and the solvent

evaporated under reduced pressure to afford 4-formyl-3-nitrobenzoic acid as a light yellow solid. Yield: 1.02g (87%). ^{1}H NMR (500 MHz, $CDCl_3$): δ 8.00 (d, J = 7.8 Hz, 1H), 8.40 (d, J = 7.8 Hz, 1H), 8.52 (s, 1H), 10.32 (s, 1H, -CHO), 14.01 (s, -COOH). FT-IR (KBr pellet, cm^{-1}): 1768 (w), 1721 (s, $\nu_{C=O}$, -CHO), 1699 (s, $\nu_{c=o}$, -COOH), 1613 (w), 1530 (s, $\nu_{N=O}$, $-NO_2$), 1415 (w), 1349 (s, $\nu_{N=O}$, $-NO_2$), 1282 (m), 1263 (m), 1119 (w).

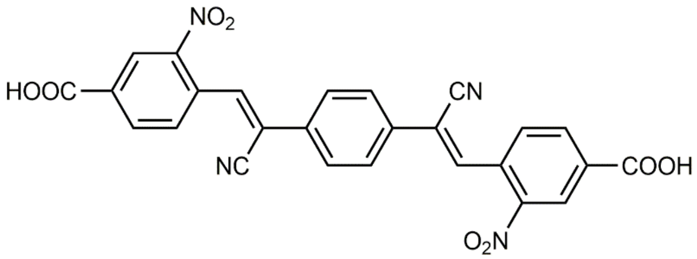

4,4'-((1Z,1'Z)-1,4-phenylenebis(2-cyanoethene-2,1-diyl))bis(3-nitrobenzoic acid) (PV3_CN_NO_2_CO_2H). 780 mg (4 mmol) 4-formyl-3-nitrobenzoic acid was dissolved in 20 mL *tert*-butanol at 50 ℃. 4 mL tetrabutylammonium hydroxide (TBAH, 1M in methanol) was added to neutralize the acid. 312 mg (2 mmol) 1,4-phenylenediacetonitrile was added and the yellow solution was stirred at 50 ℃ for 1 h. Subsequently, additional TBAH (0.4 mL, 1M solution in methanol) diluted by 2 mL ethanol was introduced dropwise into the mixture to initiate the coupling reaction, with the color of the mixture gradually changing from yellow to dark green. After stirring for 1h, the mixture was acidified by 2N hydrochloric acid to generate a yellow precipitate, which was centrifuged and washed with ethyl acetate. A yellow solid was obtained after drying overnight. To completely transform carboxylate residue to acid, the yellow solid was dispersed in 10 mL dimethylformamide and 2N hydrochloric acid was added until the dark green suspension completely changed to yellow. 50 mL water was subsequently added for further precipitation. The precipitate was filtered, washed with water and dried, and identified as the

target product. Yield: 320 mg (31%). ^{1}H NMR (500 MHz, DMSO-D$_6$): δ 7.97 (s, 4H), 8.15 (d, J = 8.0 Hz, 2H), 8.42 (d, J = 8.2 Hz, 2H), 8.57 (s, 2H), 8.66 (s, 2H), 13.88 (s, 2H) (Supplementary Figure 2a). ^{13}C NMR (500 MHz, DMSO-D$_6$): δ 165.6, 148.1, 141.6, 134.9, 134.4, 134.1, 133.8, 132.2, 127.6, 126.1, 116.7, 114.9. FT-IR (KBr pellet, cm^{-1}): 2225 (m, $\nu_{C≡N}$, -CN), 1708 (s, $\nu_{C=O}$, -COOH), 1619 (s), 1534 (s, $\nu_{N=O}$, -NO_2), 1489 (m), 1423 (s), 1348 (s, $\nu_{N=O}$, -NO_2), 1308 (s), 1266 (s), 1205 (m), 1158 (w), 1128 (w), 1073 (w). HRMS (m/z): $[M]^+$ calcd. for $C_{26}H_{13}N_4O_8$, 509.0734; found 509.0728.

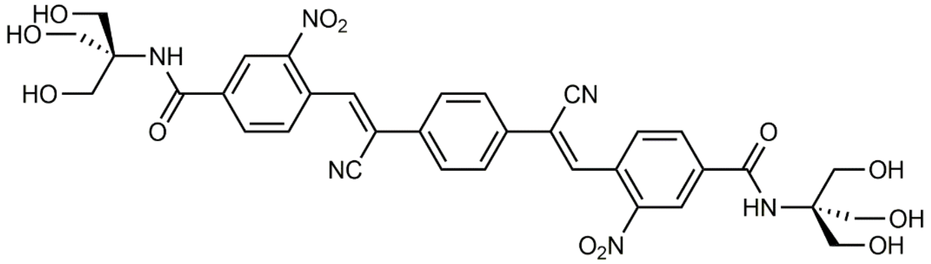

4,4'-((1Z,1'Z)-1,4-phenylenebis(2-cyanoethene-2,1-diyl))bis(N-(1,3-dihydroxy-2-(hydroxymethyl)propan-2-yl)-3-nitrobenzamide) (PV3_CN_NO_2_tripod). 31.6 mg (0.062 mmol) PV3_CN_NO_2_CO_2H, 22.4 mg (0.186 mmol) tris(hydroxymethyl)aminomethane and 56.5 mg HBTU (*N,N,N′,N′*-tetramethyl-*O*-(1*H*-benzotriazol-1-yl)uronium hexafluorophosphate, 0.149 mmol) was degassed in a 10 mL Schleck flask for 2 h, then 5 mL dry dimethylformamide (DMF) containing 0.1 mL N,N-diisopropylethylamine was syringed into the Schleck flask. The mixture was stirred at 40 ℃ for 1 h until extra 22.4 mg tris(hydroxymethyl)aminomethane was added. The reaction continued for 12 h at 40 ℃, and the product was precipitated by adding 20 mL ethyl acetate, after which the precipitate was collected in a fritted funnel, washed with distilled water and dried overnight to afford a yellow solid as the final product. Yield: 28 mg (64%). ^{1}H NMR (500 MHz, DMSO-D$_6$): δ 3.74 (d, J = 5.2 Hz, 12H), 4.68 (t, J = 6.0 Hz, 6H, -OH), 7.78 (s, 2H, -

NH-), 7.97 (s, 4H), 8.11 (d, J = 8.1 Hz, 2H), 8.33 (d, J = 8.4 Hz, 2H), 8.59 (s, 2H), 8.64 (s, 2H) (Supplementary Figure 2b). FT-IR (KBr pellet, cm^{-1}): 2225 (m, $\nu_{C≡N}$, -CN), 1724 (m, $\nu_{C=O}$), 1653 (s, $\nu_{C=O}$), 1621 (s), 1528 (s, $\nu_{N=O}$, -NO_2), 1487 (w), 1465 (w), 1416 (w), 1400 (w), 1347 (s, $\nu_{N=O}$, -NO_2), 1297 (w), 1260 (m), 1208 (w), 1146 (w), 1114 (w), 1049 (s), 1018 (w).

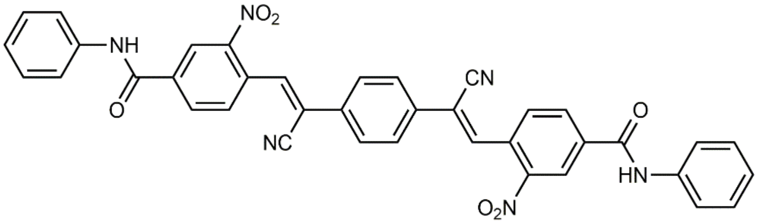

4,4'-((1Z,1'Z)-1,4-phenylenebis(2-cyanoethene-2,1-diyl))bis(3-nitro-N-phenylbenzamide) (PV3_CN_NO_2_aniline) Similar method as PV3_CN_NO_2_tripod: 31.6 mg PV3_CN_NO_2_CO_2H and 56.5 mg HBTU was degassed in a 10 mL Schleck flask for 2 h, then 5 mL dry dimethylformamide containing 0.1 mL N,N-diisopropylethylamine and 34 μL aniline (0.372 mmol) was syringed into the Schleck flask. The mixture was stirred at 40 ℃ for 12 h. and the product was precipitated by adding water, then the precipitate was filtrated by fritted funnel, washed with distilled water and dried over night to afford a yellow solid. Yield: 31 mg (76%). ^{1}H NMR (500 MHz, DMSO-D_6): δ 7.19 (t, J = 7.0 Hz, 2H), 7.43 (t, J = 7.0 Hz, 4H), 7.83 (d, J = 6.6 Hz, 4H), 8.01 (s, 4H), 8.22 (d, J = 7.0 Hz, 2H), 8.51 (d, J = 7.3 Hz, 2H), 8.63 (s, 2H), 8.82 (s, 2H), 10.71 (s, 2H) (Supplementary Figure 2c). FT-IR (KBr pellet, cm^{-1}): 2223 (m, $\nu_{C≡N}$, -CN), 1651 (s, $\nu_{C=O}$), 1621 (m), 1598 (s), 1540 (s), 1530 (s, $\nu_{N=O}$, -NO_2), 1500 (m), 1484 (w), 1445 (s), 1413 (w), 1397 (w), 1337 (s, $\nu_{N=O}$, -NO_2), 1330 (s), 1257 (s), 1260 (m), 1204 (w), 1166 (w), 1076 (w), 1028 (w).

Assignments of infrared modes of wire molecules

As shown by the comparison of the FT-IR traces (1) and (2) of Figure 3a, the only modes associated with the TMSA amine group that underwent significant red shifts upon anchoring are the NH_2 scissoring mode at 1643 cm^{-1} by 15 cm^{-1} and the CN stretch at 1303 cm^{-1} by 27 cm^{-1}, which is consistent with the change of environment of this group upon surface attachment[2,3]. With all other modes unchanged, this indicates that the silyl aniline remained intact upon anchoring. To confirm attachment of PV3 to the TMSA anchor, we examined both solid PV3 with aniline end groups and PV3 attached to TMSA on Pt/SnO_2 by infrared spectroscopy and XPS as presented in Figure 3a traces (3) and (4), and Supplementary Figure 5. By referencing the FT-IR spectra of different PV3 variants (Supporting Figure 5), we can assign many peaks in the solid PV3 sample (Figure 3a, trace (3)): the 1622 cm^{-1} band is the amide I (C=O stretch) mode, the 1600 cm^{-1} peak is the aromatic CC bond mode (overlapping with CC stretch of TMSA), and the 1533 and 1347 cm^{-1} bands are the ν_{as} and $\nu_s(NO_2)$ stretch modes, respectively[2,3]. All of these bands were also present in the IRRAS spectrum of the PV3 attached to TMSA on Pt/SnO_2 (Figure 3a, trace (4), blue shading), confirming that the *oligo*-para(phenylene vinylene) backbone and appendant NO_2 groups of the PV3 molecule are present on the Pt/SnO_2 surface. The appearance of a band at 1730 cm^{-1} in this spectrum (Figure 3a trace (4)) also indicates the terminal CO_2H is present. While the IRRAS measurement after embedding in silica was substantially more challenging than before SiO_2 deposition due to strong perturbation of the baseline caused by the silica layer (Figure 3a, trace (5)), four PV3 bands were detected, as can be seen in trace (6) of Figure 3a; C-H out-of-plane bending mode at 850 cm^{-1}, nitro group stretch modes at 1347 cm^{-1} and 1533 cm^{-1}, carboxylic acid band at 1680 cm^{-1}. It is noteworthy that the much higher intensity of the bands recorded with p polarized compared to s polarized infrared light (Supplementary Figure 6A) indicates that the wire molecules possess predominantly

perpendicular orientation relative to the SnO_2 surface[4].

Assignments of XPS spectra of embedded molecular wires

In addition to the 406.2 eV N 1*s* band of the nitro group observed upon attachment of the PV3 wire to TMSA anchored on Pt/SnO_2 (Figure 3b, trace (3)), two overlapping components at 399.69 eV (blue) and 399.44 eV (red) which arise from nitrile and amine groups[5] are seen, and a shoulder at 402.59 eV assigned to shake-up involving intramolecular charge transfer between the molecule π system (donor) and functional nitro groups (acceptor), or the PV3 $\pi \rightarrow \pi^*$ transition[6]. Ellipsometry shows that on top of the 4.7 nm SnO_2 layer is an organic layer that is 0.6 ± 0.25 nm thick, which corresponds to the TMSA height of 0.6 nm, consistent with perpendicular orientation relative to the surface. Taken together, these infrared and XPS analyses confirm that the two-step anchoring method results in the attachment of the intact wire molecules on the inorganic oxide material.

Supplementary Figures

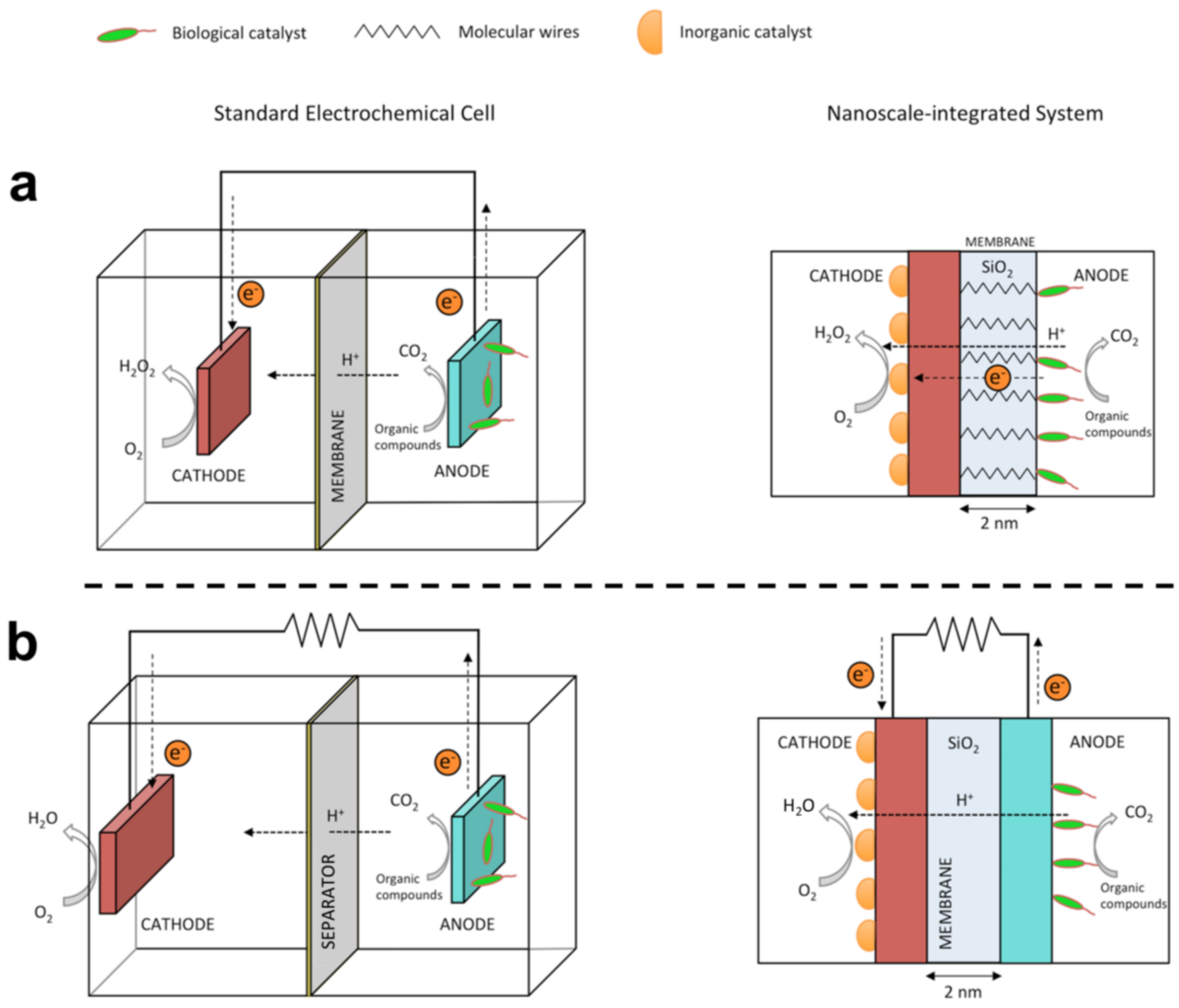

Supplementary Figure 1: Bioelectrochemical systems. **a** Microbial electrolysis cell: Comparison of macroscale coupling of electrodes (left) with nanoscale integrated cell (right). **b** Current collection for power production: Comparison of macroscale coupling of electrodes (left) with nanoscale integrated cell (right). Note that the ultrathin silica membrane is O_2 impermeable.

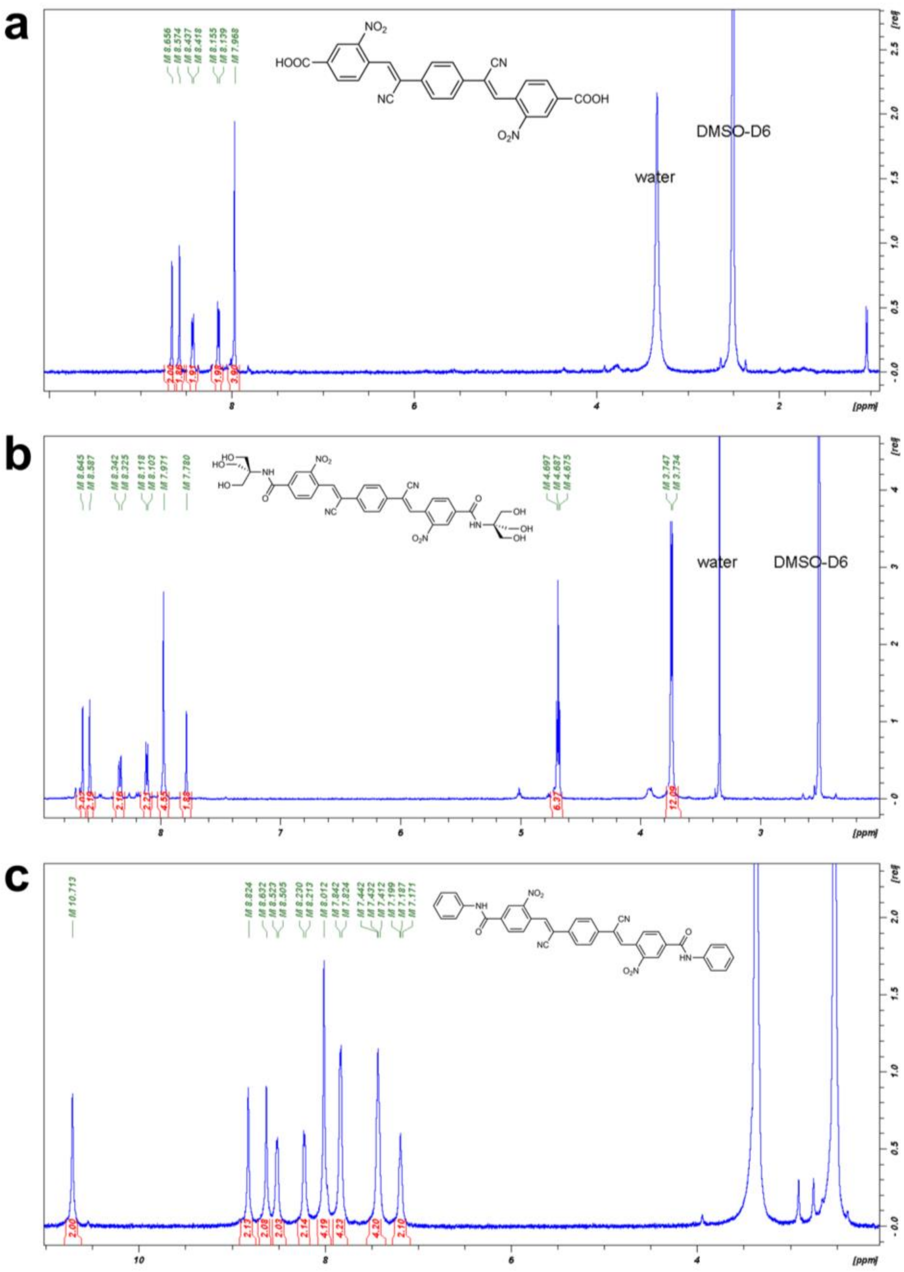

Supplementary Figure 2: a H^1 NMR spectra of PV3_CN_NO_2_CO_2H; **b** PV3_CN_NO_2_tripod; **c** PV3_CN_NO_2_aniline.

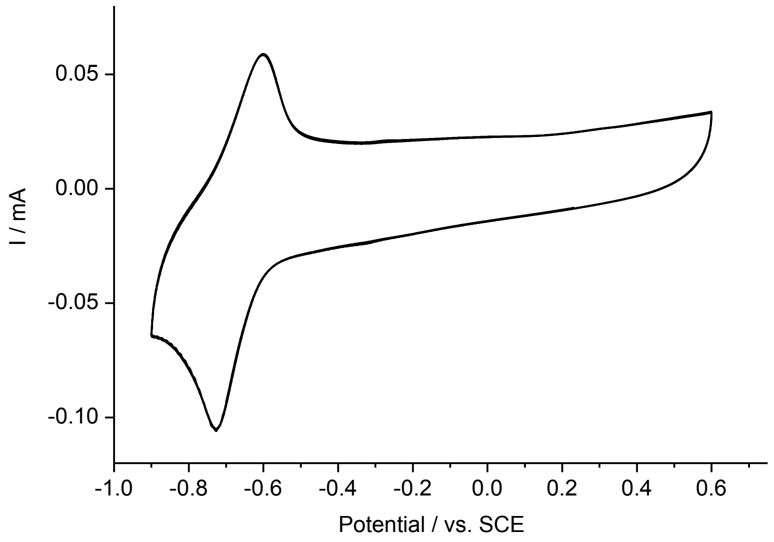

Supplementary Figure 3: Cyclic voltammogram of free PV3_CN_NO_2_CO_2CH_3 (1 mM) in Ar-purged dry dimethylformate, scan rate: 100 mV/sec, electrolyte: 0.1 M N(*t*-Bu)$_4$$PF_6$ (*t*-Bu = tertiary butyl). Working electrode: graphite rod; counter electrode: Pt. For reasons of solubility, the dimethyl ester of the compound instead of the diacid was used.

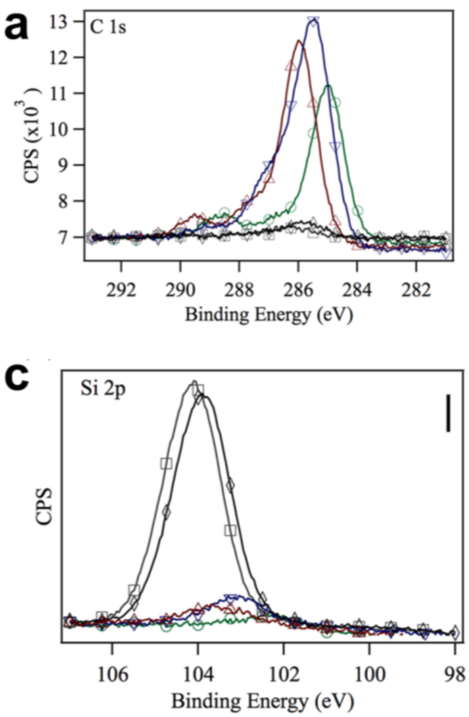

Supplementary Figure 4: XPS of carbon, oxygen, and silicon regions. Pt/SnO_2, green circles. Pt/SnO_2/TMSA, red triangles. Pt/SnO_2/PV3_CN_NO_2_CO_2H attached to TMSA, blue pointing down triangles. Pt/SnO_2/PV3_CN_NO_2_CO_2H attached to TMSA encapsulated in SiO_2, black diamonds. Pt/SnO_2/SiO_2, gray squares. Uncorrected data. **a** C 1*s* signal, shows that the signal dominates for samples containing organics TMSA and PV3_CN_NO_2_CO_2H molecules. **b** O 1*s* band, strongest for samples containing SiO_2 layer. **c** Si 2*p* band, dominates for samples with SiO_2 layer. CPS: counts per second. Scale bar 1000 CPS.

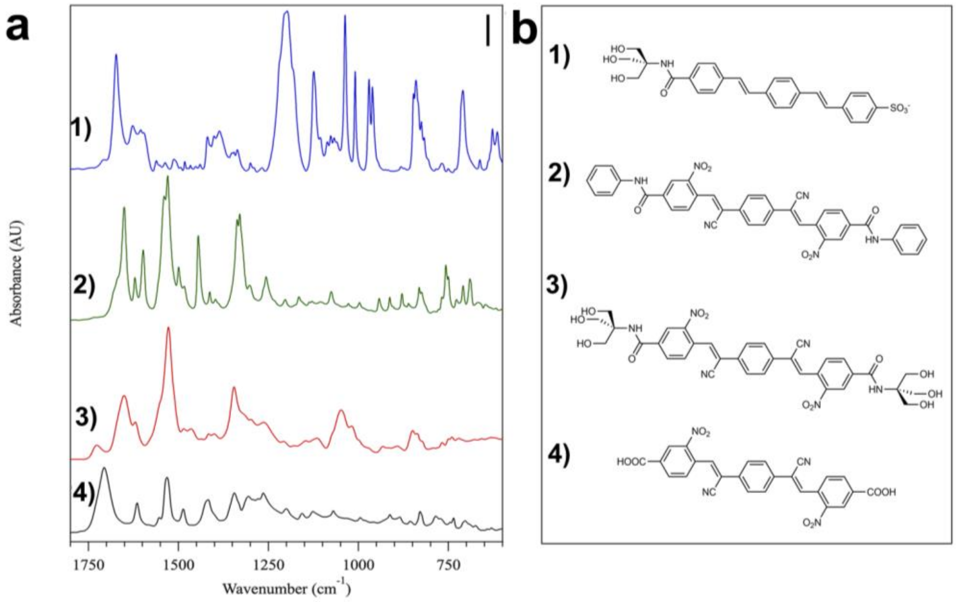

Supplementary Figure 5: FT-IR spectra of functionalized PV3 wire molecules as powders of pure compounds in KBr. **a** Absorbance spectra of (1) PV3_SO_3^-_tripod. (2) PV3_CN_NO_2_aniline. (3) PV3_CN_NO_2_tripod. (4) PV3_CN_NO_2_CO_2H. Scale bar 0.002 absorbance units. **b** Chemical structure of compounds.

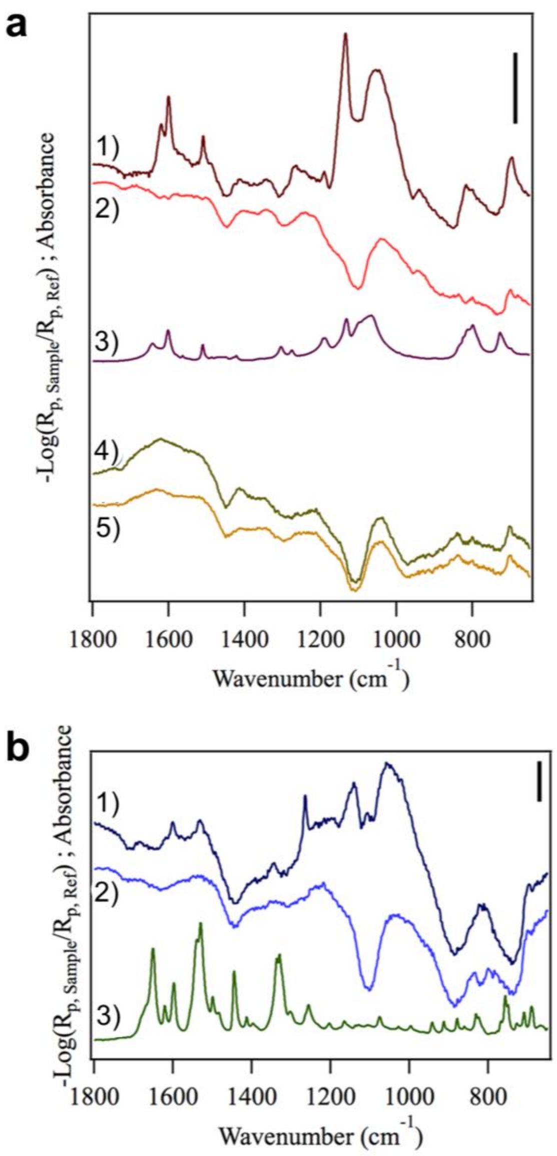

Supplementary Figure 6: FT-IR spectra of PV3_CN_NO_2_CO_2H wires attachment via TMSA linker to SnO_2 surface. **a** (1) p-polarized IRRAS of TMSA attached to Pt/SnO_2, ratioed against Pt/SnO_2. (2) Same sample, s-polarized. Resolution 2 cm^{-1}. (3) Transmission spectrum of TMSA powder in KBr. Resolution 4 cm^{-1}. (4) p-polarized IRRAS of Pt/SnO_2, ratioed against Pt. (5) Same sample, s-polarized. Scale bar 0.005. Taking into account the SnO_2 features shown in trace (4), excellent agreement is noted for the spectra of surface anchored TMSA (trace (1)) and pure TMSA (trace (3)). **b** (1) p-polarized IRRAS of PV3_CN_NO_2_CO_2H attached to TMSA on Pt/SnO_2, ratioed against Pt/SnO_2. (2) Same sample, s-polarized. (3) Absorbance spectrum of PV3_CN_NO_2_aniline powder in KBr. Scale bar 0.002.

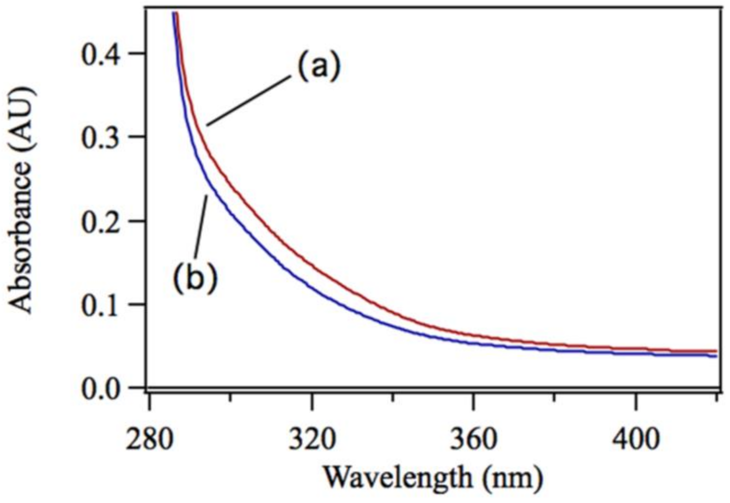

Supplementary Figure 7: UV-vis spectra of (a) PV3_CN_NO_2 anchored on SnO_2 and cast into SiO_2. The SnO_2 was deposited on a fused quartz slide. (b) SnO_2 with SiO_2 layer on quartz. To enable measurement in transmission mode, slides were immersed in dimethylformamide for index matching.

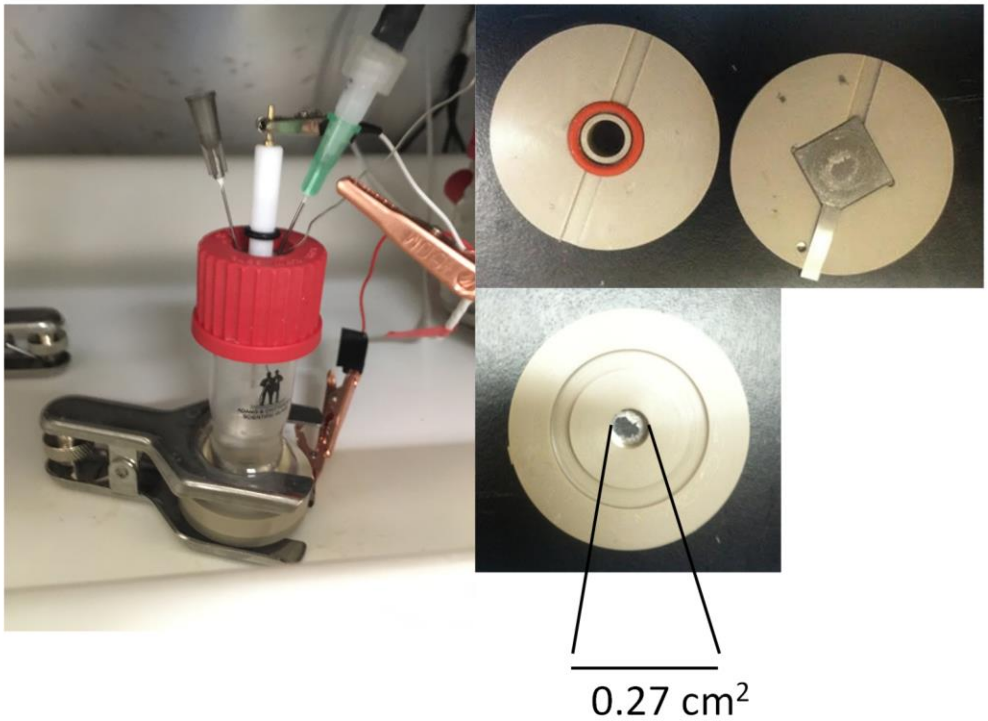

Supplementary Figure 8: Left: Electrochemical cell used for chronoamperometric measurements. The setup consists of a 10 mL reactor designed to operate in three-electrode configuration. The Ag/AgCl reference electrode and the Pt counter electrode are shown at the top of the reactor. The working electrode is located at the bottom of the reactor, fixed by a mounting support. Right: Assembly of the mounting support and the working electrode. The electrode sits in between the two discs and in contact with a metallic latch for conductivity and data acquisition and then attached to the reactor with a metal clamp.

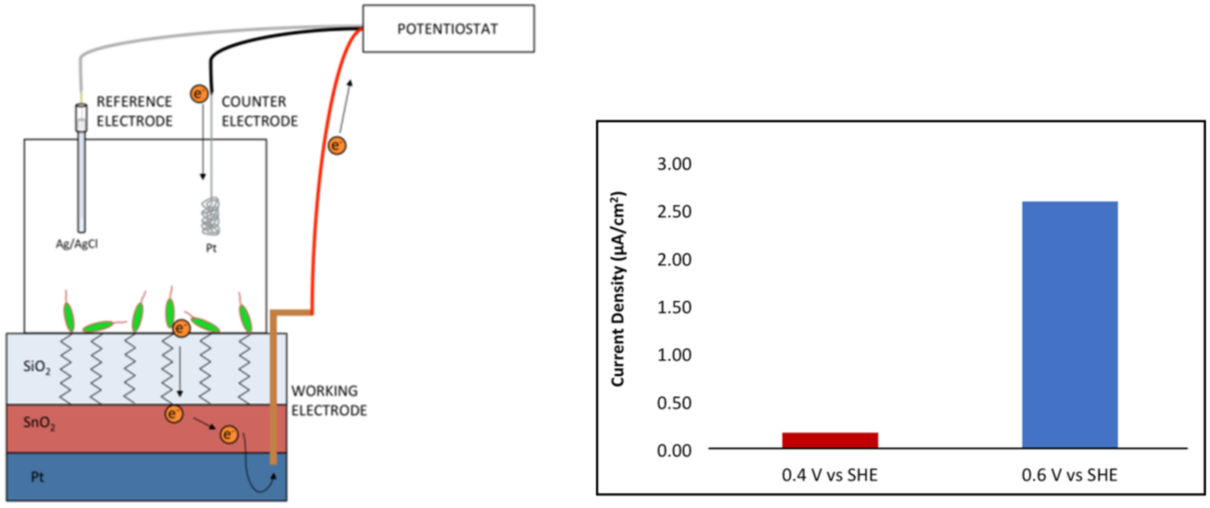

Supplementary Figure 9: 3-Electrode cell configuration for bacterial current measurements under 600 mV bias. Hydrogen is produced at the Pt cathode. The graph shows observed current for 400 and 600 mV bias (bacteria on bare SnO_2).

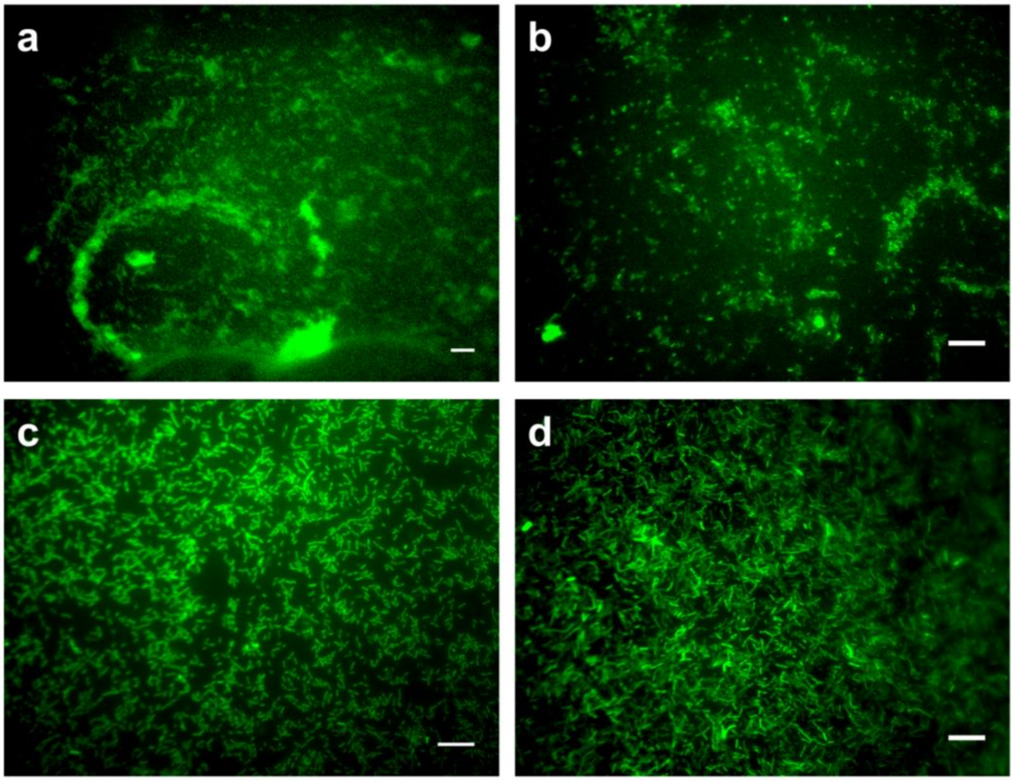

Supplementary Figure 10: Confocal fluorescence microscopy images measured from immersed surfaces in petri dishes containing *S. oneidensis* in solution. These are presented on **a** Pt/SnO_2, **b** SiO_2-encapusulated PV3 on Pt/SnO_2, **c** pure SiO_2 on Pt/SnO_2, **d** SiO_2-encapusulated PV3_SO_3^- on Pt/SnO_2. Scale bar 50 µm.

Supplementary References

[1] March, J. *Advanced Organic Chemistry*, 4th ed. (Wiley, New York, 1992; p. 945).

[2] Colthup, N. B., Daly, L. H. & Wiberley, S. E. *Introduction to Infrared and Raman Spectroscopy*, 3rd ed. (Academic Press, New York, 1990; p. 340).

[3] Bellamy, L. J. *The Infrared Spectra of Complex Molecules*, 3rd ed., Vol. 1 (Chapman and Hall, London, 1975; p. 287).

[4] Chabal, Y. J. Surface infrared spectroscopy. *Surf. Sci. Rep.* **8**, 211-357 (1988).

[5] *High Resolution XPS of Organic Polymers*, The Scienta ESCA300 Database; Wiley Interscience: New York, 1992. p. 200-202 and Appendix 4.

[6] Katrib, A., El Issa, B. D., Budeir, R. N. & Potts, A. W. The He(I) and X-ray photoelectron spectra of some substituted benzalmalonitrile compounds. *J. Electron Spectrosc. Rel. Phenom.* **49**, 233-245 (1989).
